# Respiratory muscle training positively affects vasomotor response in young healthy women

**DOI:** 10.1371/journal.pone.0203347

**Published:** 2018-09-25

**Authors:** Angela Valentina Bisconti, Michela Devoto, Massimo Venturelli, Randall Bryner, I. Mark Olfert, Paul D. Chantler, F. Esposito

**Affiliations:** 1 Department of Biomedical Sciences for Health, Università degli Studi di Milano, Milan, Italy; 2 Department of Neurosciences, Biomedicine and Movement Sciences, University of Verona, Italy; 3 School of Medicine, Division of Exercise Physiology, West Virginia University, Morgantown (WV), United States of America; 4 IRCCS Galeazzi Orthopedic Institute, Milan, Italy; University of Western Australia, AUSTRALIA

## Abstract

Vasomotor response is related to the capacity of the vessel to maintain vascular tone within a narrow range. Two main control mechanisms are involved: the autonomic control of the sympathetic neural drive (global control) and the endothelial smooth cells capacity to respond to mechanical stress by releasing vasoactive factors (peripheral control). The aim of this study was to evaluate the effects of respiratory muscle training (RMT) on vasomotor response, assessed by flow-mediated dilation (FMD) and heart rate variability, in young healthy females. The hypothesis was that RMT could enhance the balance between sympathetic and parasympathetic neural drive and reduce vessel shear stress. Thus, twenty-four women were randomly assigned to either RMT or SHAM group. Maximal inspiratory mouth pressure and maximum voluntary ventilation were utilized to assess the effectiveness of the RMT program, which consisted of three sessions of isocapnic hyperventilation/ week for eight weeks, (twenty-four training sessions). Heart rate variability assessed autonomic balance, a global factor regulating the vasomotor response. Endothelial function was determined by measuring brachial artery vasodilation normalized by shear rate (%FMD/SR). After RMT, but not SHAM, maximal inspiratory mouth pressure and maximum voluntary ventilation increased significantly (+31% and +16%, respectively). Changes in heart rate variability were negligible in both groups. Only RMT exhibited a significant increase in %FMD/SR (+45%; p<0.05). These data suggest a positive effect of RMT on vasomotor response that may be due to a reduction in arterial shear stress, and not through modulation of sympatho-vagal balance.

## Introduction

According to the American Heart Association, cardiovascular disease is the primary cause of death in the United States, [1] and the incidence of mortality due to cardiovascular events of the female population has clearly exceeded that of males since 1984. [1, 2] Exercise training is known to be cardiovascular protective [1] via a direct impact on vasculature and autonomic response. [3, 4] The beneficial effects of physical activity on the cardiovascular system are based, in part, on an indirect capacity to modify cardiovascular risk factors, such as blood pressure, lipid profile, insulin resistance, and obesity, as well as direct effects on cells and tissues of the heart and circulatory system. [5] For instance, physical exercise acts directly on the vasculature via repetitive exposure to hemodynamic stimuli, such as increases in shear stress and transmural pressure. Consequently, regular physical exercise promotes structural and functional adaptations to the vascular wall, providing putative reduction in cardiovascular risk factors.[4] However, factors, such as age, disability status, presence of injuries, and even urbanization can make physical activity difficult in many individuals, begging the question of how to improve/maintain proper physiological functioning and health in these individuals.

Repeated exercise sessions can improve vascular function by a shear-dependent mechanism, i.e., increases in blood flow on the vascular wall. [6] For instance, dynamic handgrip training has been observed to improve brachial artery vascular function in young healthy individuals. [7] Evidence also suggests the cycling exercise not only improves vascular function in the leg but also in the arm. [8] In that, cycling induces an increase in peripheral blood flow during acute exercise, thus increasing the shear stress in both the exercising legs and non-exercising arms. [8] The improvements in brachial artery vascular function, despite the lack of forearm exercise, suggest that the beneficial effects of exercise training on vascular health may be widespread and not specific to the vascular bed of the trained skeletal muscle. [9]

Respiratory muscles are part of the musculoskeletal system, and they too can be trained. [10–12] Therefore, respiratory muscle training (RMT), could be an alternative exercise paradigm and alter peripheral hemodynamics in other non- exercising areas to improve vascular heath. Indeed, there is evidence showing that RMT can increase cardiac vagal tone, [13] and leads to respiratory function enhancements such as an increase in lungs static and dynamic volumes, together with maximal inspiratory pressure, [11, 14–16] however the effects of RMT on the overall vasomotor response (a well-recognized marker of cardiovascular health) has not been investigated yet. [17]

Vasomotor response is related to the capacity of the vessel to maintain the homeostasis of the vascular tone, ensuring that the blood flow matches the demand of the skeletal muscles and other organs, both at rest and during exercise.[18, 19] Vasomotor response is regulated by the interaction of two main control mechanisms: i) the autonomic control of the sympathetic neural drive (central or global control) [18, 20] and ii) the capacity of the endothelial cells to respond to mechanical stress by releasing vasoactive factors, such as nitric oxide (NO) and acetylcholine (peripheral control). [21–23] The interaction between these two mechanisms, together with other factors, such as pH and temperature, determines the prevalence of a vasoconstriction or vasodilator effect on the arterial wall. [20, 21] Thus, an impaired vasodilatory response could be the consequence of either an autonomic and/or an endothelium dysfunction.[3, 18]

From a central/global perspective, sympatho-vagal balance can provide indirect information on sympathetic neural drive and can be assessed by heart rate variability (HRV), [24] which is the physiological phenomenon of variation in the time interval between heartbeats. In particular, the low frequency (LF) band of HRV reflects the sympathetic component, while the high frequencies (HF) are more closely related to vagal activation. HRV is affected by several factors, such as circulating hormones, modifications in heart rate and/or breath frequency, presence of pathologies, and exercise training programs. [24] It is well recognized that the balance between LF and HF can be reduced by endurance exercise training via an increase in vagal-related indices at rest, during, and immediately after exercise. [25]

At the peripheral level, the most common, non-invasive means to assess endothelial function in humans is via the flow mediated dilation (FMD) technique, [17, 26] which determines the dilation capacity of the artery in response to hyperaemic-induced shear stress. [17, 26] FMD hyperaemic response is sensitive to and strictly dependent on the frictional or drag force representing the shear stress. [22] To better determine the FMD response, either the vasodilatation, as the percentage change from baseline diameter, and the magnitude of the stimulus imposed are generally assessed. Therefore, a normalization is then applied by dividing the peak of percentage change by the amount of stimulus achieved during the reactive hyperaemia.[22] FMD response has been proven to improve after chronic exercise training. [27, 28]

Thus, the aim of this study was to evaluate the effects of eight weeks of RMT on both central (HRV) and peripheral (FMD) components of vasomotor response, in young healthy females. Our goal was to test whether RMT can improve FMD in the brachial artery (beneficial effect on peripheral control due to systemic factors influenced by exercise training) via a reduction in sympathetic drive (central control).

## Materials and methods

### Participants

Twenty-four young healthy female participants (Table 1), who were recreationally active (but not exercised trained), volunteered for study and were randomly assigned to two groups: respiratory muscles training group (RMT, n = 12) and sham group (SHAM, n = 12). The inclusion criteria included: (i) regular menstrual cycles (26 to 35 days) for at least 3 months prior to start of the study, (ii) being clinically healthy, (iii) free of cardiovascular disease, (iv) free of medications, including hormonal contraceptives and oral supplements, and (v) normal lung function as determined via medical screening and functional assessments. Each participant signed an informed consent after being fully informed on the purpose of the study and experimental procedures. The West Virginia University Institutional Review Board approved the study (protocol #1404270654), which was performed in accordance with the principles of the 1964 Declaration of Helsinki.

**Table 1:** Participants’ characteristics.

	RMT	SHAM	*P* value
Participants (n.)	12	12	-
Age (years)	25 ± 8	29 ± 9	0.27
Stature (m)	1.67 ± 6.01	1.66 ± 8.00	0.70
Body mass (kg)	64.6 ± 10.4	61.5 ± 9.9	0.47
Body mass index (kg/m^2^)	21 ± 3	19 ± 3	0.47

RMT, Respiratory muscle training. Data are expressed as mean ± SD.

### Study design

Before and after RMT, participants were tested at about the same time of the day, and at the same day of the menstrual cycle (early luteal phase) in a climate-controlled laboratory (constant temperature of 20 ± 1°C and relative humidity of 50 ± 5%). Participants documented their menstrual cycle in a personal diary throughout the study, which was used to assess the early luteal period. Subjects fasted overnight, abstained from caffeine and other similar beverages for 12 hours, and did not participate in heavy exercise for 48 hours. Post interventional experiments were performed 24 to 48 hours after the last RMT session to prevent examination of the acute effects of RMT. Moreover, an extra visit was designed for a subgroup of eight participants, who were randomly selected, in order to quantify the possible increase in brachial artery blood flow and in other central parameters, such as blood pressure, cardiac output and heart rate during a single session of respiratory muscle exercise. These data were collected after the pre-screening tests and before starting the RMT program.

### Measurements

#### Heart rate variability (HRV)

HRV analysis was used to determine the balance between the sympathetic and parasympathetic nervous system. [24, 29] Subjects laid supine for approximately 10 minutes after which the electrocardiogram (ECG) signal was recorded for other 10 minutes. Use of the ECG signal interface with the BIOPAC MP150 data acquisition (BIOPAC, ECG100C Santa Barbara, CA USA) and AcqKnowledge software allowed for advanced analysis using multiple applications. Subsequently, data were exported and analysed with Kubios HRV software (ver. 2.2, available on http://kubios.uef.fi.) that selected the clearest 5 min waves trace of the entire recording.[24, 30] ECG signal was analysed, from the distribution of the frequencies between 0.01 and 0.4 Hz. Low Frequency (LF, between 0.04 and 0.15 Hz), domain represented sympathetic nervous drive activity and baroreceptor control,[24] while High Frequency (HF, between 0.15 to 0.4 Hz) domains represented vagal activity. Given that the two branches of the autonomic nervous system operate in a coordinated approach, changes in the amount of HF and LF can provide a numerical index of the direction and magnitude of reciprocal alteration in sympatho-vagal balance. [31] Therefore, the LF/HF ratio was also calculated (as an index of the sympatho-vagal balance).

#### Flow-mediated dilation (FMD)

MD measurements of the brachial artery were performed according to recommended procedures. [32] Before FMD, the subjects laid supine for approximately 20 minutes, and then a blood pressure cuff was placed on the forearm immediately distal to the olecranon process to provide a stimulus for forearm ischemia. A 12- to 14-MHz linear array transducer, attached to a high-resolution ultrasound machine (Vivid I, GE Medical Systems, Milwaukee, WI, USA) was used to image the brachial artery in the distal one-third of the upper arm. When an optimal image was obtained, the probe was held stable and longitudinal B-mode images of the lumen-arterial wall interface were acquired. Continuous Doppler velocity assessments were also obtained using the ultrasound and were collected using the lowest possible insonation angle (always <60°). Following baseline assessments, a forearm blood pressure cuff was inflated to 250 mmHg for 5 min. Diameter and flow recordings resumed 30 s prior to cuff deflation and continued for 2 min post-deflation in accordance with recent technical specification. [32–34] The FMD data were exported in AVI format and analysed using commercially available software (Brachial Artery Analyser for Research, Medical Imaging Applications, LLC, Coralville, IA), which is largely independent of investigator bias. Furthermore, these data were verified by an expert operator blinded towards the experimental condition (RMT *vs* SHAM and pre *vs* post). After the measurements of the baseline and peak diameter, it was verified that the upper limit of 95% of confidence interval (C.I.) of the regression slope (for the logarithmically transformed baseline and peak diameter) was equal or higher than 1. [35]

FMD was quantified as the maximal change in brachial artery diameter after cuff release, expressed as a percentage increase (%Δ) above baseline:

(Peak–baselinediameter)/baselinediameter·100.

Brachial artery blood flow was estimated by using analysed software data of mean blood velocity (*v*_mean_) and arterial diameter as:

$$Blood\;flow\;(ml\cdot {min}^{-1})=v_{mean}\cdot \pi \cdot (vessel\;diameter/2)²\cdot 60$$

The shear rate (SR) was calculated post cuff release using the following equation:

$$SR\;(s^{-1})=8\cdot v_{mean}/vessel\;diameter$$

The cumulative SR, corresponding to the reactive hyperaemia post cuff release (total SR from cuff release to time to peak), was integrated (area under the curve, AUC) by using the trapezoidal rule, and calculated as:

Ʃ[yi·(x·(i−i)−xi)+(1/2)·(y·(i−i)−yi)(x·(i−i)−xi)]

The cumulative/integrated SR (AUC) reflects the amount of mechanical stimulus applied on the endothelium during the cuff release hyperaemic response at time to peak. Considering that FMD is primarily dependent on the endothelium response to mechanical stimuli, the %FMD was therefore divided by cumulative SR (%FMD/SR). [22]

#### Pulmonary function

Before and after RMT, participants underwent a pulmonary function evaluation via spirometry dynamic lung volumes (forced vital capacity, FVC; forced expired volume at 1 second, FEV_1_; and maximal voluntary ventilation, MVV) were obtained. Residual volume (RV) was also determined using a nitrogen washout method [36] and total lung capacity (TLC) was calculated as the sum of RV+ FVC. Maximal inspiratory pressure (MIP) was measured at the mouth using a portable manometer equipped with a mouthpiece (S&M Instrument Company Inc., mod. PortaResp, Doylestown, PA). After familiarization with the manometer, participants were asked to inspire as deep as possible following a normal expiration. They repeated the manoeuvre three times and the highest value was considered for comparisons before and after RMT.

#### Respiratory muscle training (RMT)

Before the first session of RMT, a sub-group of participants (n = 8) reported to the laboratory for an additional session of respiratory muscle exercise to assess the acute effects of exercise on brachial artery blood flow, SR and central parameters such as heart rate, stroke volume and mean arterial pressure. During this session, continuous Doppler brachial blood velocity and vessel diameter were assessed by ultrasound. Heart rate (bpm) and mean arterial pressure (MAP, mmHg) were determined by electrocardiography and by finger photoplethysmography (Finometer PRO, Finapres Medical System, Amsterdam, The Netherlands) positioned at the heart level, respectively. Stroke volume (SV, ml) was automatically calculated using the Modelflow method (Finapres Medical System), with cardiac output (CO, l · min^-1^) calculated as the product of SV and heart rate. The additional session consisted of two bouts of one-minute of respiratory muscle exercise performed at two different intensities: the volume was set at 50% of FVC for both intensities while the frequency was set at 20 and 30 breaths per minute during the first and second bout of exercise, respectively. A 3-minute recovery period was given between the two bouts. Before any exercise, a baseline measurement of 30 seconds was recorded. The brachial blood velocity and brachial artery diameter assessments were then used to calculated blood flow (ml · min^-1^) and SR (s^-1^). Brachial vascular conductance (BVC) was also calculated as the ratio between blood flow and MAP: BVC (ml· min^-1^· mmHg^-1^) = brachial blood flow / MAP. All the parameters were average over a time window of 30 seconds during baseline and the last 10 seconds of each recording.

**Fig 1:**
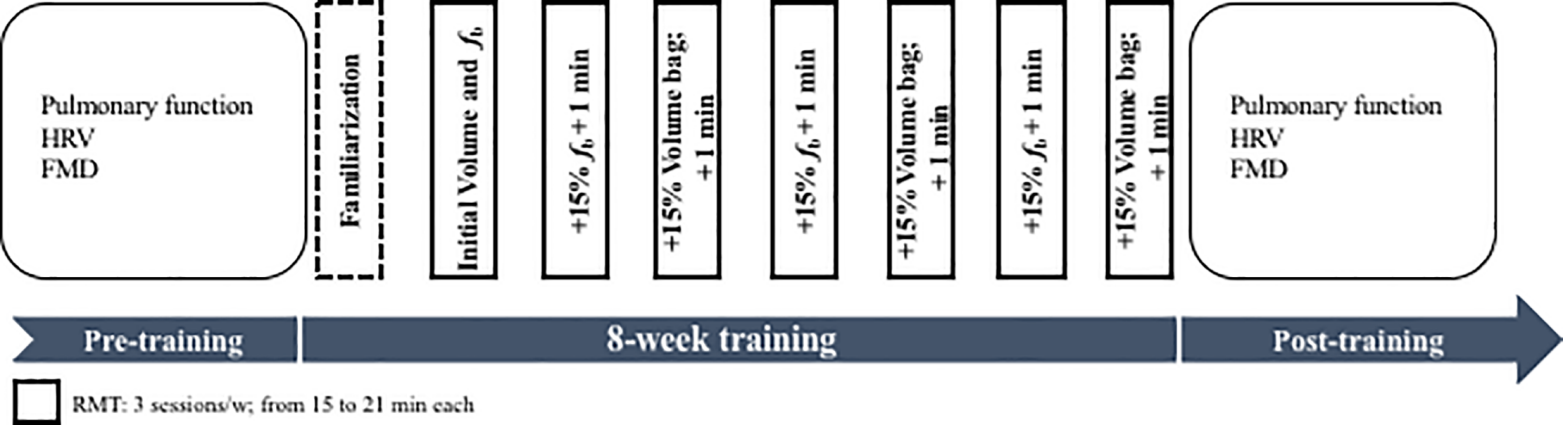
A schematic drawing of the study design.

RMT was performed for 8 weeks, 3 sessions per week for 15–30 min each, which included a warm-up and cool-down using a SpiroTiger® device (SpiroTiger® Medical, Idiag AG, Fehral- torf, Switzerland) allowing for isocapnic hyperpnoea (Fig 1). During the first week, participants were familiarized with the instrumentation and did specific respiratory muscle stretching. Starting values of the volume and frequency of respiratory cycles were determined as a percentage of FVC and MVV. In particular, the initial volume was set at 50% of FVC, while initial breath frequency (*f*_b_) was calculated as follows. [37, 38]

fb=MV/(1.3·0.5·FVC)

where respiratory minute volume (MV) was set at 60% of MVV.

The workload was increased by ∼ 15% every week by alternating either volume or *f*_b_. If necessary, small adjustments were applied according to participants’ adaptability. The RMT was monitored by an expert operator. The properties of the training device allowed personalized respiratory training through maximal inspirations and expirations. To avoid hypocapnia due to hyperventilation, the device features a two-way piston valve connected to a rebreathing bag. As the subject breathed out through the mouthpiece, the rebreathing bag stored part of the expired air, which contained increasing concentrations of CO_2_. Once the rebreathing bag was filled to its capacity, a valve opened and allowed the rest of the expired air to be released into the environment. The valve shut when expiration finished and inspiration began. Inspiration emptied the rebreathing bag first (containing increased concentrations of CO_2_), then the valve opened and some fresh air from outside was inspired at the end of each inspiration. This apparatus allows for respiratory cycles with high frequency while maintaining isocapnic hyperpnoea. The familiarization week represented the placebo-training paradigm of SHAM for the following seven weeks, for a total of eight weeks of SHAM intervention.

### Statistical analysis

Statistical analysis was performed using a statistical software package (IBM SPSS Statistics v. 22, Armonk, NY, USA). Shapiro-Wilk test was used to check the normal distribution of the sampling. A sample size of 12 healthy women was selected to ensure a statistical power higher than 0.80 based on a preliminary study on the difference between the two groups in SR. A two-way, mixed model Intraclass Correlation Coefficient (ICC) and the Standard Error of Measurements calculation as a percentage (SEM%) were utilized to assess inter-session reliability. ICC values were considered very high if >0.90, high if between 0.70 and 0.89, and moderate if between 0.50 and 0.69. The sensitivity in detecting the differences between the two trial sessions was checked by calculating the minimum detectable change at 95% confidence as a percentage (MDC95%) [39]. We assessed the possibility to express FMD in percentage as the ratio between the maximal change in brachial artery diameter and baseline diameter. A linear regression analysis between the logarithmically form of baseline and peak diameter in RMT and SHAM pre-and post- training, with the upper 95% of confidence interval (CI), was performed. [35] The upper 95% CI >1 was considered the main condition. This relationship was found to be linear and therefore we expressed FMD as a percentage (%FMD). Additionally, as explained above, %FMD was also normalized by cumulative SR (%FMD/SR). For this reason, the mean percentage difference with lower and upper CI was calculated also for RMT-induced changes in cumulative SR (AUC).

To assess significant effects of RMT on pulmonary function, HRV, and FMD parameters, a paired Student’s t-test was applied within each group, while a Student’s t-test was used to assess the differences between groups. Two separate two-way ANOVAs for repeated measures (training x time) were applied to disclose possible differences in the blood flow kinetics post cuff release during FMD. A two-way ANOVA (group x time) was utilized to determine possible differences between groups in the blood flow kinetics post cuff release during FMD. When appropriate, the post hoc Holm-Sidak test was utilized for the location of the difference. Statistical significance was accepted at P<0.05. Unless otherwise stated, values are expressed as mean ± standard error (SE).

## Results

At baseline, there were no significant differences in subject demographics between RMT and SHAM groups (Table 1).

During the two single bouts of RMT (n = 8) central parameters and peripheral blood circulation changed as follow: while MAP and CO did not change within the first intensity (101 ± 3 to 108 ± 5 mmHg (+7%; P = 0.11) and 3.49 ± 0.38 to 3.68 ± 0.55 l・min^-1^ (+6%; P = 0.059)), both parameters increased throughout the second intensity (101 ± 2 to 114 ± 2 mmHg (+ 13%; P< 0.05) and 3.40 ± 0.52 to 4.28 ± 0.43 l・min^-1^ (+ 26%; P< 0.05)). Heart rate rose from 70 ± 5 to 83 ± 7 bpm (+ 18%; P<0.05) and from 74 ± 2 to 114 ± 2 bpm (+ 26%; P< 0.05), respectively. Blood flow increased within the first and the second RMT intensities, respectively (47.8 ± 0.9 to 116.9 ± 2.2 ml・min^-1^ (+145%; P<0.001) and 50.9 ± 1.6 to 173 ± 3.37 ml・min^-1^ (+241%; P<0.001)). Also, BVC increased significantly throughout the two trials (0.47 ± 0.19 to 1.08 ± 0.12 ml・min^-1^・mmHg ^-1^ (+ 129%; P<0.05) and 0.50 ± 0.19 to 1.53 ± 0.17 (+ 203%; P<0.05) ml・min^-1^・mmHg ^-1^). SR increased from 211 ± 3 to 393 ± 5 s^-1^ (+87%; P<0.001) and from 216 ± 3 to 466 ± 8 s^-1^ (+115%; P<0.001) during the first and second RMT exercise intensities, respectively. Brachial artery diameter enlarged from 0.326 ± 0.001 to 0.377 ± 0.003 cm (+15%; P<0.001) and from 0.333 ± 0.001 to 0.404 ± 0.001 cm (+21%; P<0.001) during the first and second RMT exercise bout, respectively.

### Reliability and sensitivity of the measurements

ICC and SEM% for the investigated variables are reported in Table 2. Inter-session reliability for respiratory function was very high, with ICC ranging from 0.956 to 1.00. SEM% was between 0% and 3.6% of the relative mean value. Similarly, parameters for HRV evaluation ranged from 0.940 to 0.972, for ICC with SEM% between 3.2% and 7.6%. Lastly, variables for endothelial function assessment were very high for baseline and peak diameter (ICC: 0.998–0.999, respectively) and low for SR (ICC: 0.603) with SEM% between 0.4% and 9.2%. The MDC_95%_ calculated are also presented in Table 2. All variables presented percentage post-RMT variations higher than those required by MDC_95%._

**Table 2:** Reliability and sensitivity of the main measurements.

	Parameter	Trial 1	Trial 2	ICC	SEM%	MDC_95%_
Pulmonary function	FVC (l)	4.00 ± 0.79	3.98 ± 0.79	0.999	0.6	2
	FEV_1_ (l)	3.28 ± 0.62	3.26 ± 0.68	0.999	0.6	2
	MIP (cmH_2_O)	49.83 ± 6.71	49.83 ± 6.71	1.000	0.0	0
	MVV (l·min^-1^)	127 ± 22	132± 24	0.956	3.6	10
	RV (l)	0.70 ± 0.12	0.71 ± 0.11	0.978	2.3	6
Heart rate variability	HR rest (beats·min^-1^)	66 ± 8	64 ± 9	0.940	3.2	9
	LF (n.u.)	46.20 ± 20.51	45.98 ± 19.78	0.970	7.6	21
	HF (n.u.)	53.70 ± 21.67	53.92 ± 20.63	0.972	6.6	18
Endothelial function	Baseline diameter (cm)	0.29 ± 0.04	0.29 ± 0.05	0.998	0.7	2
	Peak diameter (cm)	0.32 ± 0.04	0.31 ± 0.04	0.999	0.4	1
	Cumulative SR (s^-1^)	288894 ± 38565	297529 ± 47360	0.603	9.2	26

FVC, forced vital capacity; FEV_1_, forced expiratory volume during the first second of the test; MIP, maximal inspiratory pressure; MVV, maximal voluntary ventilation; TLC, total lung capacity; RV, residual volume; LF, low frequency; HF, high frequency; HR, heart rate; n.u., normalized unit. ICC, interclass correlation coefficient; SEM, Standard Error of Measurement; MDC, minimum detectable change. Data are expressed as mean ± SD.

### Pulmonary function

As shown in Table 3, MIP and MVV increased significantly only in the RMT (+31% and 16%, respectively; P<0.05).

**Table 3:** Pulmonary function parameters.

	RMT				SHAM			
	Pre	%Pred	Post	%Pred	Pre	%Pred	Post	%Pred
FVC (l)	4.10 ± 0.14	101 ± 3	4.15 ± 0.16	102 ± 3	3.91± 0.2	103 ± 4	3.88 ± 0.2	104 ± 6
FEV_1_ (l)	3.37 ± 0.13	97 ± 2	3.45 ± 0.13	99 ± 2	3.18 ± 0.2	98 ± 3	3.17 ± 0.2	99 ± 3
MIP (cmH_2_O)	48 ± 4		63 ± 5*†		52 ± 2		52 ± 2	
MVV (l·min^-1^)	134 ± 4	119 ± 2	155 ± 4*†	131 ± 3	121 ± 6	116 ± 6	125 ± 7	108 ± 4
TLC (l)	4.71 ± 0.14		4.74 ± 0.14		4.70 ± 0.3		4.70 ± 0.3	
RV (l)	0.61 ± 0.07	77 ± 10	0.59 ± 0.05	74 ± 8	0.79 ± 0.05	103 ± 3	0.80 ± 0.04	104 ± 4

%Pred, percent predicted; FVC, forced vital capacity; FEV_1_, forced expiratory volume during the first second of the test; MIP, maximal inspiratory pressure; MVV, maximal voluntary ventilation; TLC, total lung capacity; RV, residual volume. Data are expressed as mean ± SE. *P<0.05 *vs* Pre. RMT, respiratory muscle training group; † P<0.05 Post-RMT *vs* Post-SHAM.

### Heart rate variability

No differences in resting HR and HRV components (LF and HF) were found, as shown in Table 4. Similarly, the LF/HF ratio did not change after RMT.

**Table 4:** Heart rate variability data.

	RMT		SHAM	
	Pre	Post	Pre	Post
LF (n.u)	44.66 ± 4.90	40.69 ± 4.95	47.65 ± 6.11	47.43 ± 5.78
HF (n.u)	55.41 ± 4.89	57.64 ± 5.45	52.06 ± 6.06	52.27 ± 5.74
LF/HF	1.00 ± 0.19	0.87 ± 0.22	1.36 ± 0.36	1.28 ± 0.32
Resting HR (beats·min^-1^)	65 ± 4	64± 3	68 ± 2	65 ± 3

HRV, heart rate variability; LF, low frequency; HF, high frequency; HR, heart rate; n.u, normalized unit; RMT, Respiratory muscle training group. Data are expressed as mean ± SE.

### FMD and reactive hyperaemia assessments

**Fig 2:**
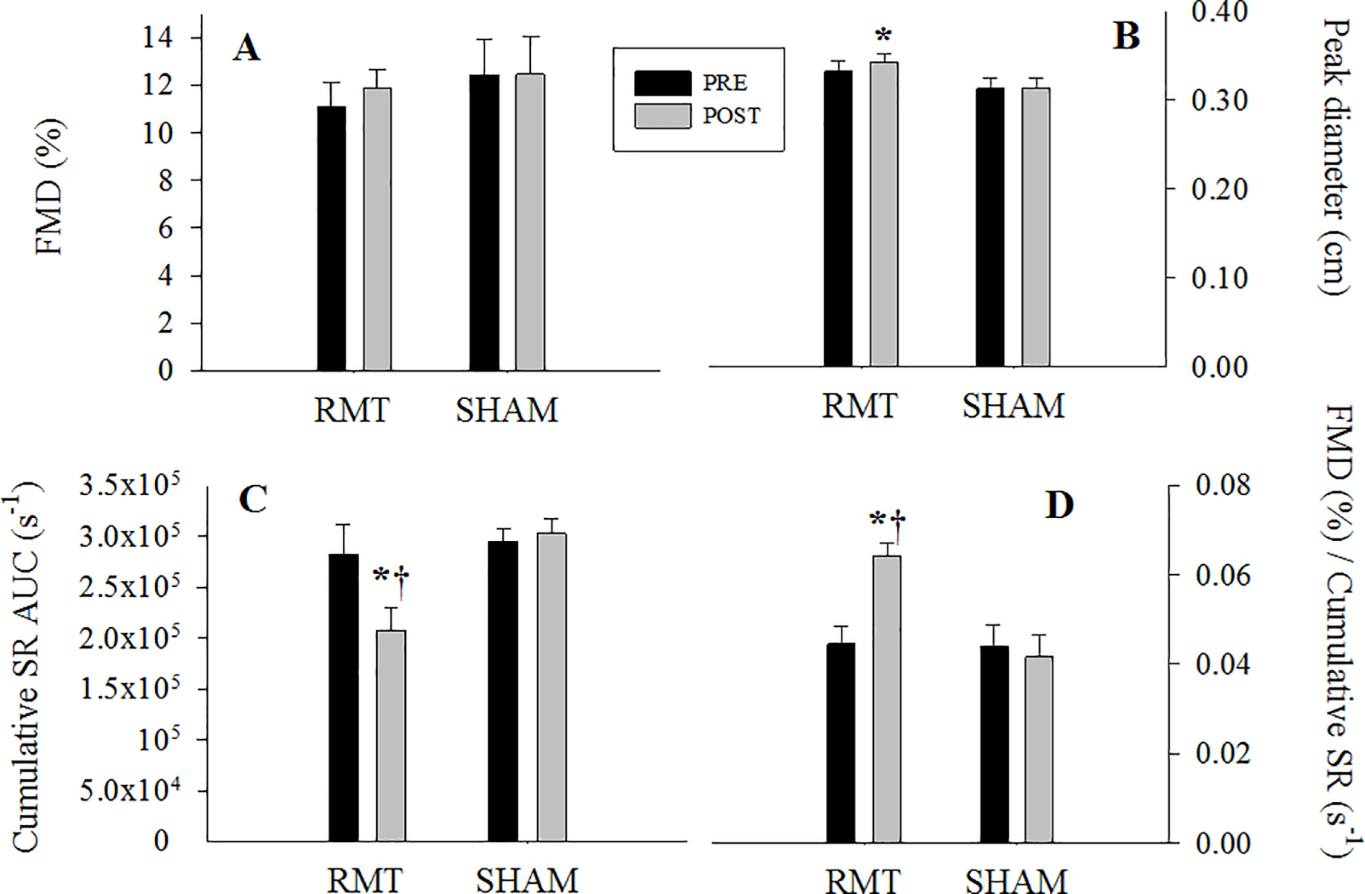
Brachial artery flow-mediated dilation (FMD), expressed as percentage change from baseline (A), absolute values at peak diameter (B), cumulative SR (C), and brachial artery FMD normalized for cumulative SR (D) are shown. RMT, respiratory muscle training. Data are expressed as mean ± SE. *P<0.05 *vs* Pre; † *P*<0.05 *vs* Post SHAM.

No differences in brachial artery diameter prior to inflation were observed, either pre-or post-training in RMT and SHAM groups (RMT: 0.30 ± 0.01cm *vs* 0.31 ± 0.01 cm, P>0.05; SHAM: 0.28 ± 0.01 cm *vs* 0.28 ± 0.01 cm, P>0.05). Although the percent change in FMD was not found to be significant (Fig 2A), a significant increase in peak brachial artery diameter following cuff release (Fig 2B) was found in the RMT group (P< 0.05). There were no differences in the time to peak diameter change between pre-and post-intervention in both groups (RMT: 35 ± 3 s *vs* 28 ± 3 s, P = n.s.; SHAM: 28 ± 2s *vs* 32 ± 3 s, P = n.s.; pre *vs* post, respectively). We found that SR was lower post-RMT (P<0.05), which was also lower than post-SHAM shear stress response (Fig 2C; P<0.05). SR percentage mean difference and CI was: 25% (-10%; 73%) and -3% (-15%; 9%) for pre- vs post- RMT and Sham comparison, whereas 41% (-55%; -3%) between group post data. Consequently, brachial artery FMD was normalized for cumulative SR (%FMD/SR) and post-RMT %FMD/SR increased by 45% (Fig 2D; P<0.05).

**Fig 3:**
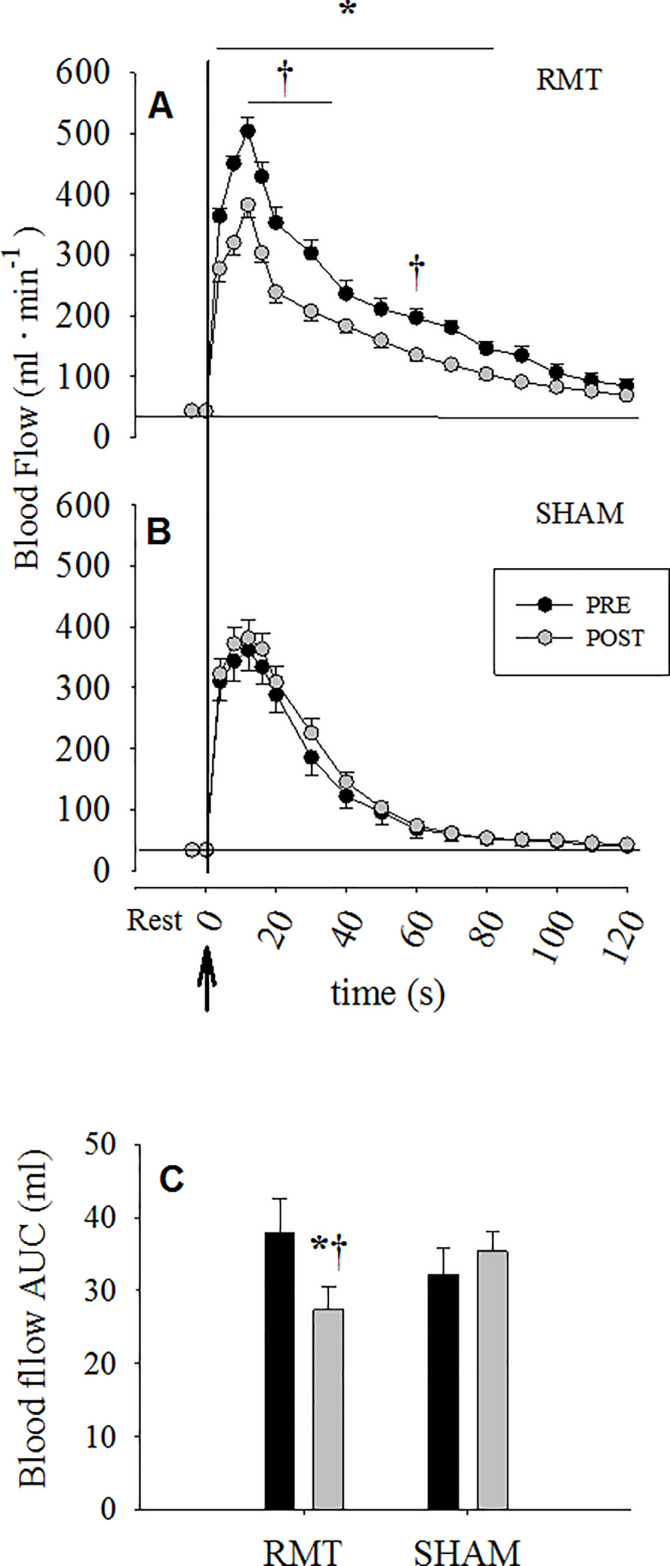
Post occlusion reactive hyperaemia, expressed as absolute blood flow (3A and 3B) and area under the curve (AUC, 3C) in RMT (respiratory muscle training) and SHAM group. The ↑ in proximity to zero represents the exact time of cuff release. Data are expressed as mean ± SE. *P<0.05 *vs* Pre. †P<0.05 *vs* Post SHAM.

Although baseline brachial artery blood flow was not different between RMT and SHAM (Fig 3A and 3B), the hyperaemic blood flow response, after the cuff release, during the FMD manoeuvre was blunted in post-RMT from second 4 to 80 after cuff release and then returned to baseline levels (Fig 3A). This response was not seen in SHAM (Fig 3B). Consequently, the blood flow AUC post-training was 29% (P<0.05) lower than pre only in RMT but not in SHAM (Fig 3C).

## Discussion

This is the first study to assess vasomotor response in young heathy women both before and after a respiratory muscle intervention, and provides an important finding the RMT can be used to improve vascular health in individuals who could otherwise have limited locomotor abilities. Indeed, RMT resulted in a significant decrease brachial artery shear stress, however, no significant differences were noted in sympatho-vagal balance. As the vasomotor response at the peripheral level is predominantly ascribed to the interaction of central neural control (assessed by HRV) and peripheral vascular control (assessed by FMD), these findings suggest that RMT enhanced vasomotor response via a shear stress, rather than to autonomic balance as the principal mechanism.

While physical inactivity, together with endothelial and autonomic dysfunction, predisposes all individuals to premature cardiovascular disease, disability, and death, [1, 3] we chose to focus this study on females given the fact that the incidence of mortality due to cardiovascular disease has dramatically exceeded that of men since the mid 1980’s. [1] Moreover there is limited availability of data on vascular function in women following RMT, and we hope to expanding this knowledge with the aim to uncover new approaches capable of preserving cardiovascular health in women and potentially be effective to reverse this negative trend. However, the RMT effects on vascular function is still an open question also in young healthy males. Indeed, due to a different hormonal state, young male individuals have a different HRV balance, resulting in a higher sympathetic activation. [40, 41] RMT could be beneficial in rebalancing HRV by enhancing parasympathetic drive over the sympathetic activation,[13] allowing the speculation that different effects of RMT could be expected in a male population.

The effectiveness of the training protocol was confirmed by the improvement in pulmonary function following RMT, as indicated by the increase in MVV and MIP. These ameliorations, which were qualitatively expected especially because the training device stressed both the inspiratory and expiratory muscles, are well in agreement with previous findings. [13, 15, 16, 37, 42, 43]

### Effect of RMT on heart rate variability

RMT has been generally utilized to improve pulmonary function, [11, 14, 15] as well as respiratory muscle endurance and performance. [16] It has been reported that RMT can also affect HRV in healthy individuals by decreasing LF power and increasing HF power. [13] As a consequence, a reduction in the sympathetic drive and possibly an increase in peripheral blood flow via a reduction in peripheral resistances may occur, similarly to what happens in heart failure and hypertensive patients. [44, 45] In a recent metanalysis, [25] endurance training (involving cycling or running) was commonly utilized to enhance vagal-related indices of HRV. Endurance training triggers a chain of reactions, among which an increase in left ventricular internal dimension and wall thickness, and in end-diastolic volume (by increasing plasma volume and decreasing peripheral resistances), leading to an increase in stroke volume that allows for a lower HR at rest. [25] This HR modification at rest is partially linked to a gradual increase in parasympathetic modulation, which is generally reflected in the HF peak of HRV analysis. [25] On the contrary, despite an augmented respiratory muscle endurance performance, our study did not result in any significant modifications in resting HR and in LF or HF. Therefore, the assumption can be made that the type of stimulus was not strong enough to induce the central adaptations mentioned above. Additionally, RMT has been shown to provide benefits somewhat similar to those induced by endurance training on vagal activity, even though resting HR does not change. [13] Hepburn et al. found not only an increase in HF component, which reflects the vagal tone, but also a reduction in LF, which mainly represents the sympathetic activation. [13] These data are not in agreement with the findings of the present investigation. However, it is important to note that some methodological differences exist between the two studies. Indeed, Hepburn and co-workers recruited an older population compared to the present study (52 ± 4 *vs* 25 ± 9 years, respectively), with different baseline values of LF and HF (LF 49.9 ± 6.2 and HF 41.7 ± 5.7 *vs* LF 44.66 ± 4.90 and HF 55.41 ± 4.89, respectively). A non-uniform impairment in sympathetic and parasympathetic tone with aging, which is generally associated with a reduction in the sympatho-vagal balance, has been widely recognized. [40, 46] Taking this into consideration, the positive RMT effects on HRV components in that study were likely derived from baseline LF and HF. Other prior studies demonstrated that RMT can positively affect sympathetic and parasympathetic activity [47] in patients with hypertension and heart failure. [14, 45] Thus, it is tempting to speculate that the positive effects of RMT on HRV may be only visible in a population already characterized by alterations of the sympatho-vagal balance. It should be also taken into account that remarkable gender differences in HRV have been previously observed. [40, 41] Specifically, males have lower HRV than females on the same age [40, 41] because of a stronger sympathetic tendency over parasympathetic activation [40, 41] due to a different hormonal situation. [48] In post-menopausal women, this gender difference is reduced. [41] Based on these considerations, we cannot exclude that RMT may lead to positive results on HRV in men via a remodelling in the autonomic activation consisting in a decrease in sympathetic neural drive. Nevertheless, further studies are required to fully elucidate this hypothesis.

### Effect of RMT on flow mediated dilation

Our findings clearly suggest an RMT-induced decrease in shear stress on the brachial artery inner during FMD, with no changes in sympatho-vagal balance, as assessed by the LF/HF ratio. Therefore, the improvement in vasomotor response after RMT could be predominately ascribed to peripheral vascular, rather than central neural adjustments.

In the present study, RMT effects on the vasomotor response are related to peripheral vascular control, as measured by endothelial cells capacity to respond to mechanical stimuli such as occlusion/hyperaemic reperfusion during FMD manoeuvre (i.e., endothelial dependent vasodilation response), are quite complicated and may need some further considerations. It is well known that vasodilation occurs in response to FMD due to an acute increase in blood flow following the release of circulatory occlusion in a limb for a short period. [32, 49] The resulting hyperaemia increases shear forces on the vessel’s wall [49, 50] and triggers a chain of reactions that lead to higher endothelial NO synthase activity and induce relaxation of the smooth muscles and subsequent vasodilation. The increase in arterial diameter following reactive hyperaemia reaches a peak value that is generally compared to baseline diameter. [32] The positive effects of training on FMD, expressed as percentage change from baseline diameter (%FMD), have been widely documented. [51, 52] Birk, Dawson [8] investigated the role of chronic shear stress stimulus on brachial artery FMD before and after eight weeks of cycle exercise training. During each training session, one arm was always partially occluded by a cuff to mitigate distal shear stress in the non-exercising district, while the other non-exercising arm was freely perfused. FMD results revealed an improved peripheral vascular response only in the free (non-occluded) arm, [8] confirming the key role of shear stress in this phenomenon. [8, 53] In the present study, %FMD did not change after RMT, even if RMT markedly increased the peripheral circulation and shear stress in the brachial artery (non-exercising district) during each RMT session. Moreover, as reported in the results section, RMT was a powerful exercise stimulus not only able to increase the amount of peripheral blood flow in the brachial artery, but also to increase all the central parameters such as MAP, heart rate, CO and BVC during a single bout of RMT. Specifically, acute RMT data revealed that, even if CO and MAP did not increase significantly during the first exercise intensity, their percentage change was higher than baseline and coupled with the significantly increase in brachial artery blood flow and SR. The second intensity was strong enough to significantly increase all the cardiovascular parameters including the peripheral hemodynamics. In the present study, the first acute intensity mimicked the same RMT intensity experienced during the first week of the training protocol. On the contrary, the second intensity aimed at mimicking the minimum intensity increment achieved by everyone throughout RMT. The large increase in BF can be primarily explained by the increase in MAP, causing the increase in BVC and therefore in shear stress.

As shown in Table 2, the cumulative SR is a relative variable parameter. Considering the difference between pre- and post- in RMT group, a moderate yet significant difference was found. Nevertheless, the mean difference in cumulative SR between post-RMT and post-SHAM values clearly highlights the efficacy of RMT treatment in readjusting the amount of mechanical stress necessary to exacerbate FMD response. However, given that FMD is related to shear stress and is proportional to reactive hyperaemia, [32] it can often be normalized to the cumulative SR. [22] This normalization process unveiled a positive effect of RMT on peripheral vasomotor response. Thus, it is possible that mechanical stimuli on the vasculature wall during RMT (i.e., the continuous SR increment in the brachial artery during the training sessions) were not as efficient as during cycle exercise training and their effects could be highlighted only after the accounting for the relative shear stress.

This scenario may be explained by several potential mechanisms, such as a reduction in reactive oxygen species, increase in NO bioavailability and/or vascular remodelling. Physical exercise and muscle contraction, indeed, lead to accumulation of reactive oxygen species. [54] For instance, diaphragm muscle, which contracts continuously to maintain respiration, could lead to an overproduction of reactive oxygen species, limiting its functionality. [55] Moreover, reactive oxygen species are important to maintain vascular homeostasis, and their extra production could impair endothelial function because of the decrease in NO bioavailability. [56] Likely, the proper intensity of regular exercise, such as RMT, could stimulate positive adaptive responses to counteract an excessive reactive oxygen species production, thus modulating muscle redox balance and NO bioavailability. [54, 56]

In addition, peak diameter increased after RMT, together with a consistent drop in cumulative shear stress, leading to an increased %FMD/cumulative shear stress. This observation suggests that after RMT the same percentage change in diameter could be achieved with a lower mechanical stimulus allowing the speculation that RMT likely led to a more responsive vessel. On this premise, despite the lack of FMD response after training in the present study, it is plausible that RMT provided a physiological stimulus (i.e., the exercise-induced increase in SR) that led not only to a more compliant system, but also to an increase in NO bioavailability in young, healthy women. Further studies are needed to better clarify the physiological pathway related to the observed drop in cumulative SR.

The positive effects observed in the present study, in terms of %FMD/SR increase, may suggest that RMT is able to raise the peripheral blood flow, which, with chronic training, triggering a series of events, [57] that lead finally to an improvement in the vascular structure and function. [58]

The implications of these findings effect of RMT may benefit the recovery of the endothelium after deconditioning, which can be useful in those populations characterized by low mobility, such as the elderly or in athletes recovering from an injury. Given that we examined the effects of RMT in women future studies are necessary to fully elucidate the effects of RMT on vascular function in men, and in chronic disease populations that are associated with altered shear stress or autonomic dysfunction.

In conclusion, eight weeks of RMT in a young female population improved vasomotor response mainly via an amelioration of the peripheral vascular control mediated by release of local vasoactive factors. Indeed, the autonomic sympatho-vagal balance (central neural control), as assessed by HRV, was not affected by RMT. On the other hand, a marked increase in the %FMD/SR was found in RMT group but not in SHAM. Such an increase was possibly due to a drop in the cumulative SR without any changes in %FMD. Future studies are needed to fully elucidate the mechanisms underpinning these RMT-induced modifications.

## Supporting information

S1 Dataset De-identified trial data. (XLSX)
